# Techno‐economic analysis of a plant‐based platform for manufacturing antimicrobial proteins for food safety

**DOI:** 10.1002/btpr.2896

**Published:** 2019-09-11

**Authors:** Matthew J. McNulty, Yuri Gleba, Daniel Tusé, Simone Hahn‐Löbmann, Anatoli Giritch, Somen Nandi, Karen A. McDonald

**Affiliations:** ^1^ Department of Chemical Engineering University of California Davis California; ^2^ Nomad Bioscience GmbH Halle Germany; ^3^ DT/Consulting Group Sacramento California; ^4^ Global HealthShare® Initiative University of California Davis California

**Keywords:** plant‐based production platforms, production of antimicrobial proteins, techno‐economic modeling

## Abstract

Continuous reports of foodborne illnesses worldwide and the prevalence of antibiotic‐resistant bacteria mandate novel interventions to assure the safety of our food. Treatment of a variety of foods with bacteriophage‐derived lysins and bacteriocin‐class antimicrobial proteins has been shown to protect against high‐risk pathogens at multiple intervention points along the food supply chain. The most significant barrier to the adoption of antimicrobial proteins as a food safety intervention by the food industry is the high production cost using current fermentation‐based approaches. Recently, plants have been shown to produce antimicrobial proteins with accumulation as high as 3 g/kg fresh weight and with demonstrated activity against major foodborne pathogens. To investigate potential economic advantages and scalability of this novel platform, we evaluated a highly efficient transgenic plant‐based production process. A detailed process simulation model was developed to help identify economic “hot spots” for research and development focus including process operating parameters, unit operations, consumables, and/or raw materials that have the most significant impact on production costs. Our analyses indicate that the unit production cost of antimicrobial proteins in plants at commercial scale for three scenarios is $3.00–6.88/g, which can support a competitive selling price to traditional food safety treatments.

## 1 INTRODUCTION

The World Health Organization estimates 600 million cases of foodborne illness worldwide in 2010, of which 420,000 resulted in death.^1^ Food safety is an alarming global challenge for human health. Food supply chains are increasingly geographically diverse, requiring coordination between multiple governments and food industry stakeholders.^2^ In the United States, surveys estimate 9.4 million cases of foodborne illness, 55,961 hospitalizations, and 1,351 deaths each year.^3^ A single foodborne illness outbreak can have significant economic impact, estimated to cost a restaurant between US $4,000 and $2.6 million.^4^ Such statistics underscore the fact that foodborne illnesses place a significant burden not only on the U.S. healthcare system at $14 billion annual cost of illness^5^ but also on key stakeholders in the food industry and our economy in general.

Current food sanitizing practices aimed at minimizing such outbreaks predominantly involve thermal inactivation or treatment of food with organic acids, salts, or ultraviolet (UV) irradiation. These treatments are largely effective, yet may still present foodborne disease vulnerability in key processing steps for many products. For example, recent literature highlights the challenge of the “viable but nonculturable” (VBNC) state of microorganisms in these food‐sanitizing treatments.^6^ One or more of the current food sanitizing treatments have been shown to induce a VBNC state from which reversion to a culturable state is possible for major foodborne disease‐associated microorganisms such as *Escherichia coli*,^7^ *Salmonella enteritidis*,^8^ *Listeria monocytogenes*,^9^ and *Shigella flexneri*.^10^

Biotic approaches to food sanitization have high potential as supplementary treatments to de‐risk the supply chain by employing efficacious and orthogonal protection against high‐risk pathogens. Food safety applications of bacteriophages (viruses capable of killing bacteria), endolysins (antibacterial proteins derived from bacteriophages), and bacteriocins (antimicrobial proteins produced by bacteria for ecological dominance) have already been approved for commercial use in the United States. For example, Intralytix Inc. offers a suite of FDA‐approved bacteriophage‐based antibacterial food safety products (ListShield™, EcoShield™, SalmoFresh™, and ShigaShield™). Human exposure to large numbers of bacteriophage and bacteriocin is likely in a typical diet as well as from commensal microflora in the gastrointestinal tract. Therefore, there is a strong and intuitive case for acceptance of certain bacteriophage‐ and bacteriocin‐derived antimicrobial treatments for food safety applications.^11^ In fact, various preparations of bacteriophages, such as the *Salmonella*‐specific bacteriophage cocktail SalmoFresh™,^12^ endolysins,^13^, ^14^ and bacteriocins, such as colicins^15^, ^16^ and nisins,^17^ have already been granted Generally Recognized as Safe (GRAS) status as food antimicrobials by the US Food and Drug Administration (FDA). It is anticipated that similar antimicrobial preparations will be granted GRAS status by FDA in the future, as the popularity of these technologies grows and additional regulatory notices are filed.

The costs of standard food sanitizing treatments are as low as $0.01–0.10/kg food.^18^ In the cost‐constrained markets of food additives and processing aids, these new biotic approaches to food sanitation will need to be accessible at the low selling prices that the food industry is accustomed to, or gain market entrance as a luxury good on the basis of their differentiating features, including worker safety in the preparation and handling of the products, environmentally friendly disposal, nonimpact on the organoleptic properties of food, and no or minimal food matrix alteration.^19^ Strategies to meet low cost of use can be broadly classified as either pertaining to molecular engineering of the treatment agent or manufacturing science and technology. Substantial research has been done to employ genetic engineering to alter the action of native antimicrobial proteins.^20^ For example, the modular structure of the bacteriophage class of enzymes known as endolysins provides a perfect “Lego® block”‐like molecular engineering platform to swap the N‐terminal catalytic domain or the C‐terminal binding domain to create novel hybrid moieties.^21^

Although molecular engineering approaches possess substantial potential for human therapeutics, changes to the native structure of antimicrobial proteins for food safety applications bar them from taking advantage of the expedited GRAS marketing allowance pathway. For antimicrobials that are novel, or altered, and hence not “generally recognized” as safe, the alternative marketing approval route (food additive petition) requires a full preclinical safety data package, which is a costly and time‐consuming process that creates a significant barrier to entry for new food safety interventions, given the above‐mentioned current pricing structures, regulations, and public perception. Consequently, biotic food safety approaches are more amenable to cost containment through manufacturing science and technology.

The cost sensitivity of the food industry is the most significant barrier to the adoption of new food sanitizing treatments, such as antimicrobial protein (AMP) preparations. Plant‐based platforms have the potential for producing market‐relevant volumes of AMPs at competitive costs, because they do not require expensive bioreactors and culture media. In recent studies, we have shown that plants such as *Nicotiana benthamiana*, spinach, and leafy beets are an attractive and scalable production platform for production of AMPs, including antibacterial colicins, salmocins, and bacteriophage endolysins. We have previously reported expression levels as high as 3 g/kg plant fresh weight (FW).^11^, ^14^, ^15^, ^22^, ^23^, ^24^ In this study, we address cost sensitivity with a comprehensive techno‐economic analysis of plant‐based production of AMP for food safety applications. We used laboratory‐scale results and working process knowledge from pilot and commercial processes to develop a process simulation model using SuperPro Designer® to assess the commercial viability of the production platform and to identify economic “hotspots” to help guide future research and development.

A selection of recently published studies on the techno‐economics of *N. benthamiana* plant‐based production of a variety of recombinant proteins is summarized in Table 1.^25^, ^26^, ^27^, ^28^ To our knowledge, this study is the first techno‐economic analysis of a plant‐based production platform for AMPs as food safety additives.

**Table 1:** A selection of recently published techno‐economic analyses of *Nicotiana benthamiana* plant‐based production models for molecular farming

Parameter	Unit	Tusé et al.^26^		Walwyn et al.^27^	Nandi et al.^28^	Alam et al.^25^
		(1)	(2)			
Industry	–	Pharmaceutical	Biofuel	Reagent	Pharmaceutical	Pharmaceutical
Molecule	–	Butyrylcholinesterase	Cellulase enzyme	Horseradish peroxidase enzyme	Monoclonal antibody	Antiviral protein
Expression system	–	Transient; agroinfiltration	Transgenic; inducible	Transient; agroinfiltration	Transient; agroinfiltration	Transient; viral vector
Production	kg/year	25	3 *×* 10^6^	5	300	20
Expression	g/kg FW	0.5	4	0.24	1	0.52
Recovery	%	20	–	54	65	70
Purity	%	>95	–	250 kU/g	>95	>99
CAPEX	$ million	92.4 (U/D 3:7)	11.5 (U/D 10:0)	–	122 (U/D 4:6)	–
COGS	$/g	1,180 (U/D 3:7)	6.9 × 10^−3^ (U/D 10:0)	1,279 (U/D 2:8)	90–121 (U/D 4:6)	105.80(U/D 6:4)

Abbreviations: BChE, butyrylcholinesterase; CAPEX, capital expenditures; COGS, cost of goods sold; HRP, horseradish peroxidase; mAb, monoclonal antibody; U/D, ratio of upstream to downstream costs.

## 2 MATERIALS AND METHODS

### 2.1 Process simulation

The plant‐based AMP production and purification process was modeled using SuperPro Designer® Version 10 (Intelligen, Inc., Scotch Plains, New Jersey; http://www.intelligen.com), a computer modeling tool capable of sizing equipment, performing material and energy balances, developing flowsheets, scheduling operations and debottlenecking. SuperPro Designer® built‐in unit models include a suite of manufacturing unit operations (>140) that can be configured to represent a manufacturing process flow diagram for the biotechnology, pharmaceutical, and food industries. The software uses these process flows and unit operations to then generate process and economic reports, including annual operating expenditures (OPEX) and capital expenditures (CAPEX). All currency is listed in USD.

The manufacturing process flow (e.g., unit operations, materials, process parameters) was developed using working process knowledge, unpublished lab‐, pilot‐ and commercial‐scale data, and data published in the literature. Built‐in SuperPro Designer® equipment design models were used for equipment sizing.

### 2.2 Host selection

*Nicotiana benthamiana* is used as the plant host organism in the base case scenario. *Nicotiana benthamiana* is used extensively for indoor plant molecular farming applications based on its rapid growth, genetic tractability, susceptibility to agrobacterium transformation, and high expression levels of recombinant proteins.^29^, ^30^, ^31^ The species is used in the commercial scale production of therapeutics and vaccines by companies such as Kentucky BioProcessing Inc. (Owensboro, Kentucky),^32^ Medicago Inc. (Québec, Quebec, Canada),^33^ and iBio CMO (Bryan, Texas).^34^

The modeled facility is designed to accommodate a previously reported process using transgenic *N. benthamiana* featuring a double‐inducible viral vector, developed by Icon Genetics GmbH (Halle/Saale, Germany). Published results demonstrate minimal background expression of recombinant protein until the induction of deconstructed viral RNA replicons from stable DNA proreplicons is triggered by 1–20% (v/v) ethanol applied as a spray on the leaves and/or a drenching of the roots, to achieve expression levels as high as 4.3 g/kg plant FW.^35^ Although the more common *Agrobacterium*‐mediated transient expression production platform enables rapid production of recombinant target molecules,^36^ this transgenic system obviates the need for additional expenses associated with *Agrobacterium tumefaciens* preparation, vacuum infiltration, and agrobacterium‐introduced endotoxin removal.^37^

### 2.3 Facility design

The simulated manufacturing facility is composed of two separate process models/flowsheets: (1) the upstream processing models the plant growth, ethanol induction, and product generation, which feeds into (2) the downstream processing model for purification of the product from the process and product impurities to meet food processing aid specification. Quality assurance (QA), quality control (QC), and laboratory costs associated with good agricultural and collection practices (GACP) for upstream processing and FDA food industry current good manufacturing practice (cGMP) for downstream processing are included in the design. Equipment, materials of construction, and prices are also modeled on food cGMP standards.^38^ The location of commercial‐scale plant molecular farming operations of Kentucky BioProcessing Inc. (Owensboro, Kentucky) was selected as the basis for location‐dependent costs. Location‐dependent costs (e.g., electricity and municipal water) are based on values obtained from publicly available Owensboro, Kentucky municipal pricing charts (https://omu.org/). The simulated manufacturing facility is assumed to be a greenfield single‐product biomanufacturing facility that is operational 24 hr per day and 7 days per week with an annual operating time of 90% or 329 days per year.

Independent market analyses project a reasonable base case facility production level of 500 kg AMP per year for food safety applications of interest (unpublished data). To meet this demand, the proposed facility employs three‐layer vertically stacked indoor plant cultivation stages designed for hydroponic host plant growth in a soilless substrate to support the plant and its roots. The cultivation stages are equipped with a light‐emitting diode (LED) lighting system and a recirculating ebb and flow hydroponic water supply. The cultivation stage plant growth is divided into a series of trays that advance unidirectionally across the plant cultivation room toward automated plant harvesters and further downstream processing. Automated belts convey harvested plant tissue to the double‐stack disintegrator and further downstream processing.

A compilation of facility and process parameter inputs is presented in Tables S1–S4 or in the base case model itself, which is publicly available at http://mcdonald-nandi.ech.ucdavis.edu/tools/techno-economics/.

### 2.4 Upstream processing

**Figure 1:**
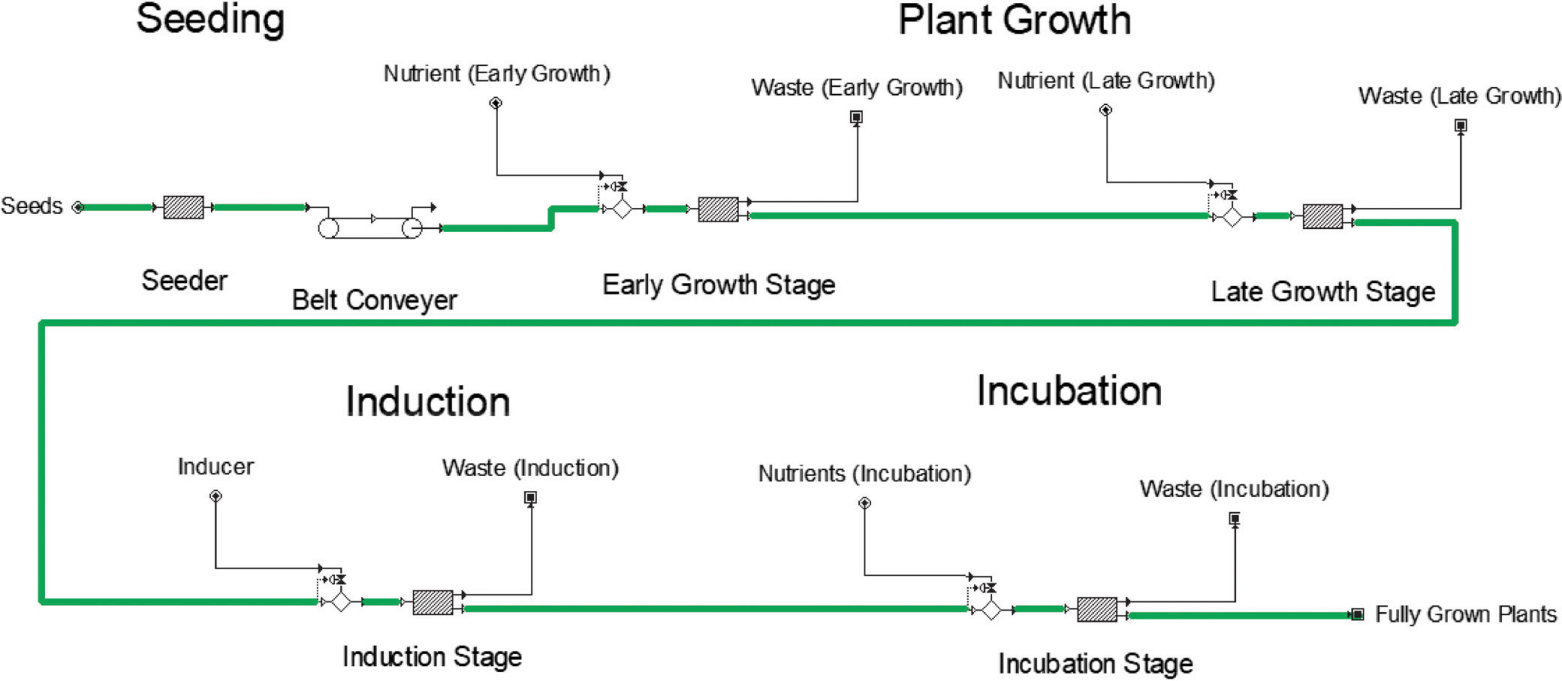
Upstream process flowsheet for the *Nicotiana benthamiana* base case scenario and *Spinacia oleracea* alternative scenario using the SuperPro Designer model

The upstream processing model flowsheet is graphically depicted in Figure 1. Transgenic *N. benthamiana* seeds consumed in upstream processing are generated in‐house from validated Working Seed Banks, which were in turn generated from validated Master Seed Banks. The seed bank release testing includes germination efficiency >95%, confirmation of growth kinetics, and viral testing. CAPEX related to seed generation are excluded, but associated seed production costs are included in the estimate of $9.50/g seed (1 g of seed is approximated as 9,500 seeds).

The seeds are set in soilless plant substrate at a density of 94 *N. benthamiana* seeds per 30 × 50 cm tray. The seedlings are cultivated hydroponically during the plant growth phase to reach manufacturing maturity by 35 days. Nutrient solution for plant growth is recirculated with minimal waste and routinely monitored and adjusted for consistent quality based on pH and conductivity. At manufacturing maturity, the plants are transferred to an induction space, complete with a separate hydroponic reservoir, curtains for temporary enclosure, and double rail spray booms. Recombinant expression of AMP is induced over the course of 1 hr via root drenching and aerial tissue spraying with a combined 0.01 L of 4% (v/v) ethanol per kg FW plant tissue. The plants are then moved to the incubation phase. Post‐induction plants are expressing recombinant AMP, and so the nutrient solution is circulated via a separate feed tank and hydroponic reservoir. The nutrient solution in the incubation phase may contain trace levels of ethanol, which may prematurely initiate AMP production and impair plant growth kinetics. AMP accumulates in the *N. benthamiana* tissue over the course of 6 days. The nutrient solution in the incubation phase is not recirculated between batches, but sent to biowaste instead, amounting to an overall 23% plant uptake of the nutrient solution. The spent nutrient solution in the incubation phase is treated as biowaste to address trace amounts of the viral expression vector that may be present in solution.

### 2.5 Downstream processing

**Figure 2:**
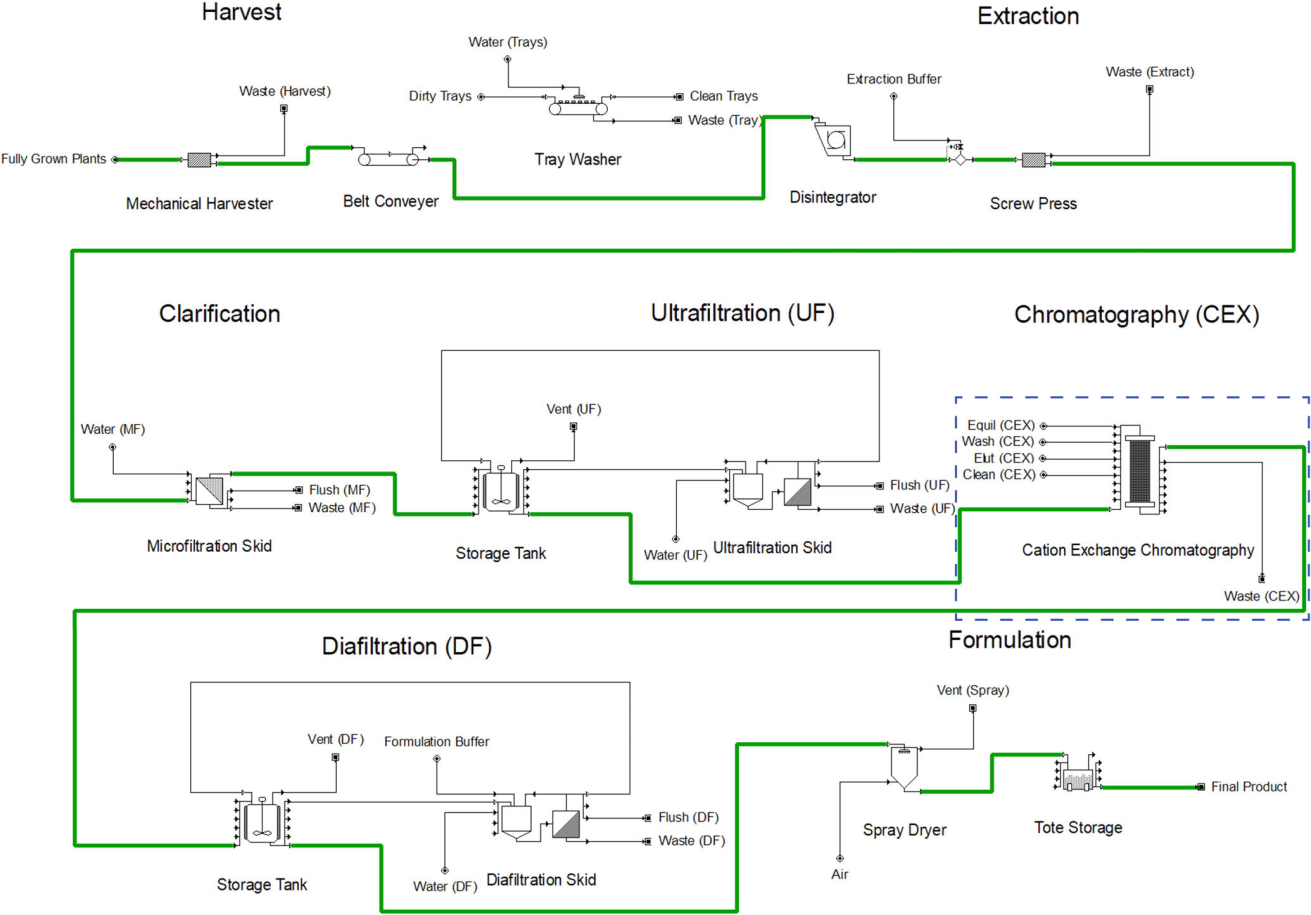
Downstream process flowsheet for the *Nicotiana benthamiana* base case scenario and *Spinacia oleracea* alternative scenario in the SuperPro Designer model. The chromatography step, outlined in dotted blue, is only in the *N. benthamiana* base case model

The downstream processing model flowsheet is graphically depicted in Figure 2. Downstream processing begins with plant harvest. This starts with automated harvester collection of aerial *N. benthamiana* plant tissue. The spent soilless plant substrate is sent to waste along with the remaining *N. benthamiana* root matrix. The disposal costs for this step are considered negligible and are not explicitly calculated in the model. There are several routes possible for disposal of plant growth substrate such as composting on site, using it for mulch on facility landscape, collection by farmers for spreading on agricultural land, and, as a last resort, sending it to a landfill. It may be possible, and more cost effective, to sterilize and reuse the growth media but this was not considered in the model. The harvested trays are cleaned in an automated washer with 0.1 L of water per tray. The harvested plant tissue is conveyed via automated belts to extraction, which starts with a double‐stack disintegrator to reduce plant biomass particle size. The disintegrated tissue is then sent to a screw press with an extraction ratio of 0.5 (v/w) extraction buffer:plant FW for acidic extraction. The extraction buffer and conditions for efficient *N. benthamiana* extraction have been reported.^39^ All buffer compositions can be viewed in Table S5. A plant‐made AMP purification protocol uses similar acidic extraction to remove *N. benthamiana* host proteins.^11^

The plant extract is clarified using tangential flow microfiltration. The clarified stream is then ultrafiltered with additional tangential flow filtration using a 10 kDa molecular weight cutoff to a concentration factor of 20.

The AMP in the retentate stream is then purified with cation exchange column chromatography in a bind‐and‐elute mode of operation. The AMP is eluted isocratically in elution buffer (50 mM sodium di‐hydro phosphate, 1 M NaCl). The purified stream is subjected to one final tangential flow filtration procedure for buffer exchange into phosphate‐buffered saline (PBS) with a diafiltration factor of 3 (i.e., volume of diafiltrate buffer/volume of solution). The purified formulation is spray dried and filled in 1‐L plastic bags to obtain the final bulk AMP.

All downstream processing water in direct contact with the product stream is reverse osmosis (RO) water. All equipment from extraction to formulation are sanitized postprocessing with a clean‐in‐place (CIP) procedure consisting of a prerinse with municipal water, caustic wash with 0.5 M NaOH, postrinse with municipal water, acid wash with 0.5% (w/w) HNO_3_, and a final rinse with RO water. Storage tanks are additionally sanitized preprocessing with steam‐in‐place (SIP).

### 2.6 Scenario analysis

Base case scenario outputs were used to identify parameters with significant impact on process economics. We focused the scenario analysis on two different classes of parameters: facility performance parameters and resource purchase costs. Facility performance parameters are defined as inputs that directly impact the physical outputs of the model. Typical biotechnology facility performance parameters include host organism expression level, unit operation recovery, and yearly production level. We chose to investigate expression level and yearly production level. To analyze the impact of facility performance parameters, we set a parameter range based on working process knowledge and then developed a model (corresponding to a redesigned facility) derived from the base case scenario for each parameter increment within the range. Facility performance parameter changes result in a cascade of changes to the model inputs and outputs; each model is adapted to the resulting stream composition and throughput of the given parameter value while maintaining the constraints of the fixed base case scenario process inputs.

Resource purchase costs are defined as inputs that directly control the economic impact of resource utilization for outputs of the model. For the purpose of this analysis, purchase price parameters are contained to cost items within OPEX.

### 2.7 Alternative scenarios

Alternative facility design scenarios were developed as comparative models to more broadly explore the context of the base case scenario process economics. The alternative scenario models were designed in alignment with base case scenario inputs unless otherwise noted; each alternative scenario was chosen to isolate the impact of a key facility design assumption.

The first scenario investigates an alternative transgenic leafy plant host organism, spinach (*Spinacia oleracea*) cultivar Industra, for the base case scenario indoor growth and ethanol‐inducible expression. Some colicins have been successfully expressed in *S. oleracea* (spinach) plants; however, their expression levels were approximately 10 times lower than that in *N. benthamiana* so additional research is needed to increase production levels.^11^, ^14^, ^22^ Several salmocins and lysins can be expressed at high levels in spinach, which is comparable to expression levels in *N. benthamiana*.^17^, ^20^ The primary distinction in this alternative plant host organism is the lack of nicotine, the major alkaloid in *Nicotiana* species. In the base case scenario, significant downstream processing emphasis is placed upon nicotine removal. The upstream and downstream processing model flowsheets are graphically depicted in Figure 1 and Figure 2. A complete list of changes to the base case scenario inputs can be observed in Table S4.

The second scenario investigates outdoor field‐grown transgenic ethanol‐inducible *Nicotiana tabacum* as an alternative to an indoor plant growth facility. Large Scale Biology Corporation previously investigated *N. tabacum* outdoor field‐grown production of recombinant proteins and personnel involved in that work recommended pursuit of this agronomic approach, with special consideration of field condition variability on product consistency.^40^ *N. tabacum* is used instead of *N. benthamiana* for its increased resilience to agricultural pathogens and weather fluctuation.^40^ The upstream processing model is adapted from a techno‐economic analysis of plant‐made cellulase produced in the field.^26^ The upstream and downstream processing model flowsheets are graphically depicted in Supplementary Information, Figure S1 and Figure S2. A complete list of changes to the base case scenario assumptions can be viewed in Table S6.

## 3 RESULTS

### 3.1 Facility operation of base case

The base case manufacturing facility scenario produces 500 kg of AMP per year at 92% purity including a 42% loss in extraction, downstream processing, and formulation. This yearly production is achieved in 91 manufacturing batches, each with a 42.3‐day duration, which process 1.22 million plants (or 1.22 × 10^6^ plants) per batch with an expression level of 1 g AMP per kg plant FW for a yearly total of 111 million plants processed. The facility plant inventory is 14.7 million plants, which is divided into 12 concurrent batches of plant growth. Initialization of batches is staggered by 3.42 days. The AMP is produced and recovered through a series of manufacturing steps: plant growth, ethanol induction, incubation, harvest, extraction, clarification, concentration, chromatographic purification, buffer exchange, and formulation. The upstream processing recipe (seeding, plant growth, induction, and incubation) cycle time is 41.4 days and has been designed as the production bottleneck; the downstream processing recipe cycle time is 0.91 days and is thus executed well within the allowable stagger time between plant harvest cycles.

#### 3.1.1 Upstream processing

To meet the yearly production demand of 500 kg AMP, upstream processing must produce 867 kg AMP to offset the 42% downstream processing loss. Each upstream processing batch yields 9,520 kg *N. benthamiana* plant FW containing 9.52 kg AMP, which represents 10% of the total soluble protein (TSP).^11^ This results in 866,000 kg *N. benthamiana* plant FW processed over the course of the 91 annual batches, grown in 111,000 units of soilless plant substrate with 1.30 million liters of plant nutrient. Of the annual plant nutrient volume, 436,000 L are sent to waste while the remainder is utilized during plant growth. A total of 7,410 L of 4% (v/v) ethanol are consumed annually for induction.

#### 3.1.2 Downstream processing

Manufacturing batches continue directly from upstream to downstream processing; batches are not pooled, and thus 91 downstream processing batches are executed annually. Each batch begins with the upstream production of 9,520 kg *N. benthamiana* plant FW and 9.52 kg AMP. Screw press extraction results in a stream mass flow of 11,200 kg per batch (0.61% host impurities, and 0.08% AMP). After microfiltration (membrane area, 26 m^2^) and ultrafiltration (membrane area, 26 m^2^) the stream is considerably reduced to 476 kg per batch (0.83% host impurities, and 1.41% AMP). The product stream is eluted from the cation exchange chromatography (resin volume, 283 L) at 236 kg per batch (0.22% host impurities, and 2.48% AMP). The product stream is then diafiltered for a buffer exchanged product stream of 230 kg per batch (0.21% host impurities, and 2.38% AMP). The final spray dry formulation results in 9.06 kg formulated product per batch (5.32% host impurities, and 60.64% AMP).

### 3.2 Economic analysis of base case

**Figure 3:**
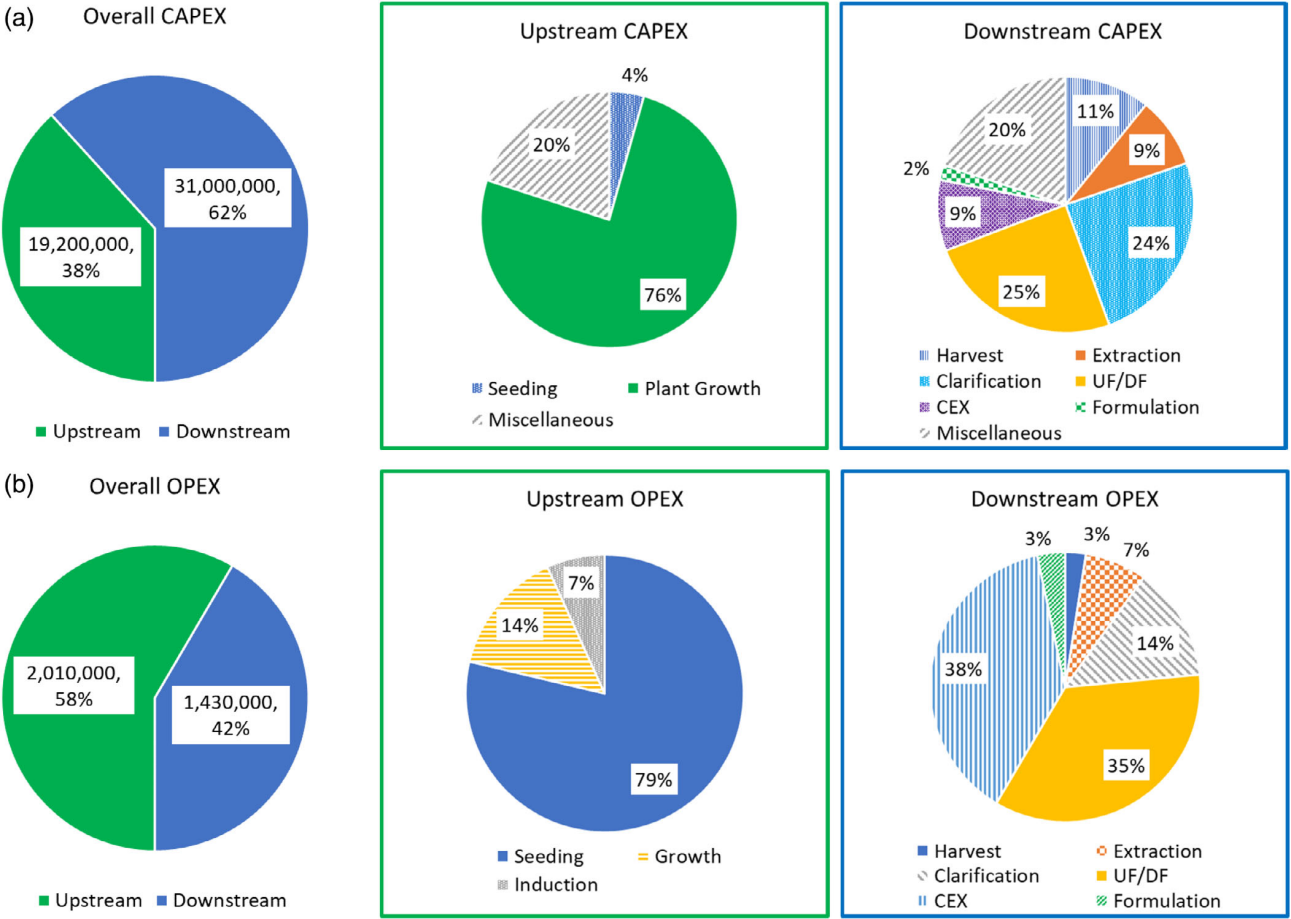
Economic assessment of upstream and downstream processing (a) operating expenditures (OPEX) and (b) capital expenditures (CAPEX) for the *Nicotiana benthamiana* base case scenario. CEX, cation exchange chromatography; UF/DF, ultrafiltration/diafiltration

The base case manufacturing facility requires $50.1 million CAPEX and $3.44 million/year OPEX. The AMPs' cost of goods sold (COGS) is calculated to be $6.88/g. Figure 3 shows an economic assessment of upstream and downstream processing. Upstream processing represents 58% of overall operating expenditures (OPEX), and downstream processing makes up the remaining 42% of operating costs. Of the $2.01 million/year upstream OPEX, the seeding operation (mainly because of the cost of the consumable soilless plant substrate) represents the majority (79%) of the cost. Chromatography (38%) and ultrafiltration/diafiltration (UF/DF) operations (35%) represent the majority of downstream processing OPEX of $1.43 million/year. The downstream CAPEX accounts for 62% of the overall CAPEX with the clarification and UF/DF filtration units representing the largest portion (49%) of the downstream capital investment costs.

### 3.3 Purchase price sensitivity analysis

**Figure 4:**
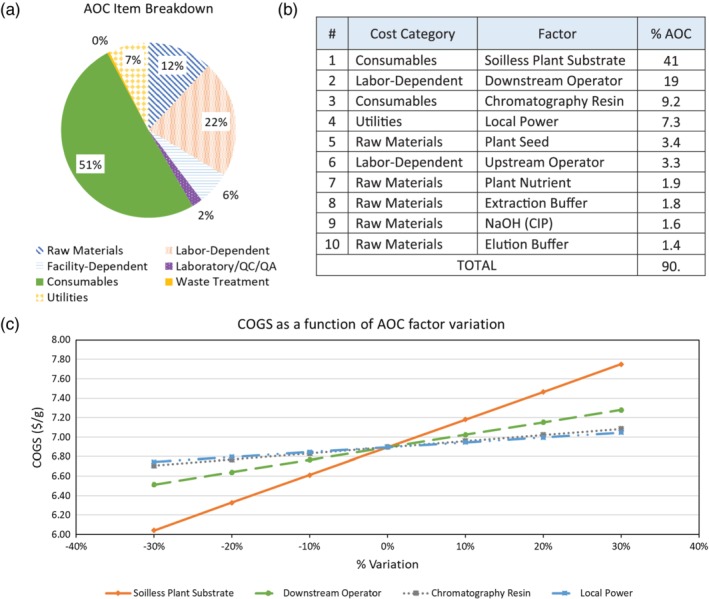
For the *Nicotiana benthamiana* base case scenario (a) annual operating cost (AOC) breakdown of the base case scenario based on cost category. (b) Top individual factors and the respective cost category, contributing to the AOC of the base case scenario. (c) Cost of goods sold (COGS) as a function of the price of the top individual factors contributing to the AOC

The annual operating costs are heavily weighted by a small number of process inputs; the top 10 cost factors collectively represent 90% of the annual operating cost. Figure 4 shows the top 10 cost factors and the impact of largest contributor percentage variation (±10, 20, and 30%) on the AMP COGS. At 1.3 cents per plant, the soilless plant substrate alone accounts for 41% of the OPEX; ±30% variation in soilless plant substrate corresponds to ±12% overall COGS. Variation of ±30% in the 10th largest contributor, the chromatography elution buffer, results in ±0.41% change in overall COGS. As expected, variation in the larger contributors to annual operating cost results in larger changes in COGS.

### 3.4 Expression level and production capacity analysis

**Figure 5:**
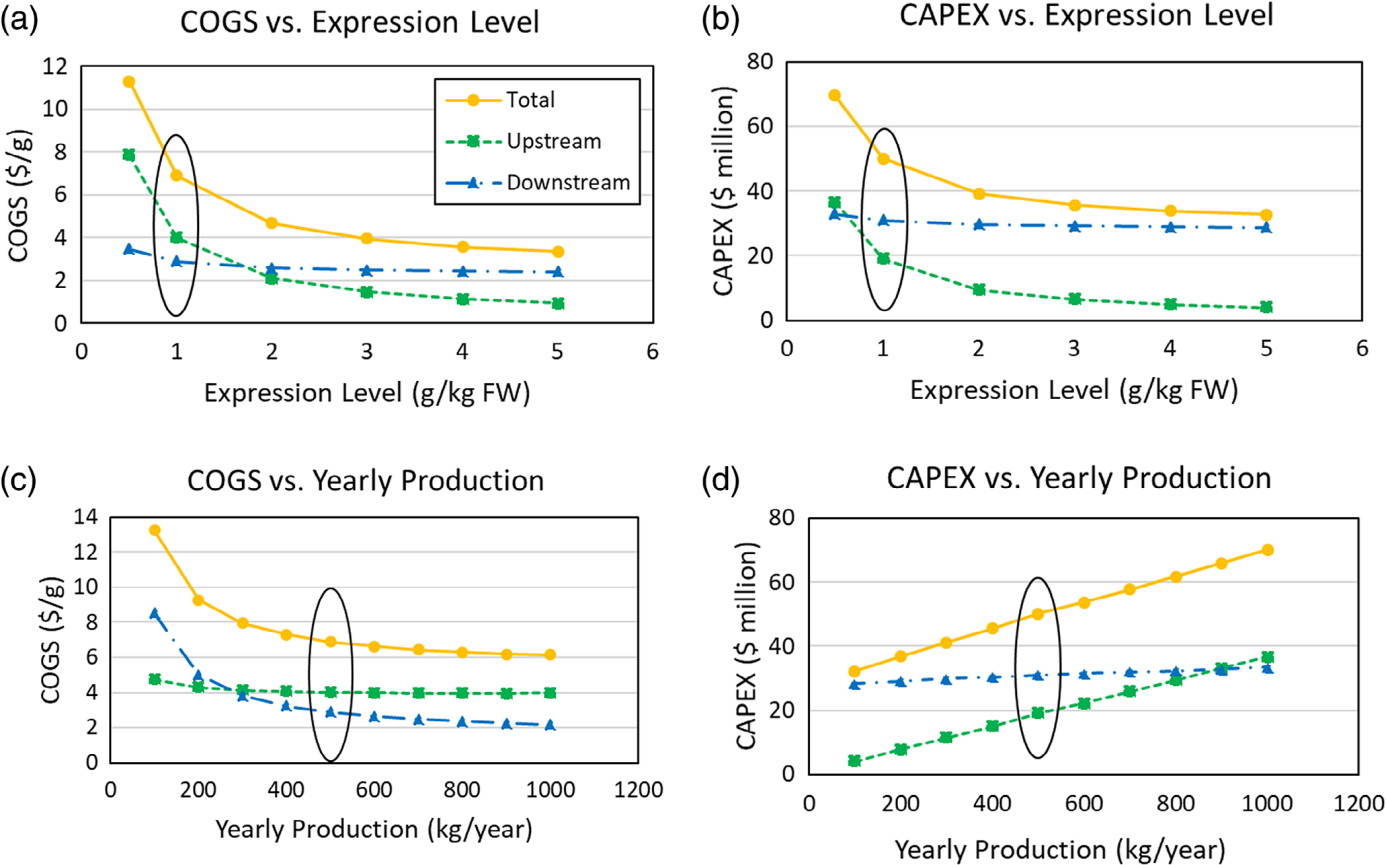
For the *Nicotiana benthamiana* scenario, analysis of expression level variation on (a) cost of goods sold (COGS), (b) capital expenditures (CAPEX), and of yearly production variation on (c) COGS and (d) CAPEX. Yearly production is fixed at the base case of 500 kg AMP/year for expression level variation analyses (a,b). Expression level is fixed at the base case value of 1 g AMP/kg FW for yearly production variation analyses (c,d). Total, upstream, and downstream contributions of COGS and CAPEX are displayed. The base case scenario values are circled in black. FW, fresh weight

To evaluate the impact of AMP expression level and facility AMP production level, we developed models for a 500 kg AMP/year production level with different AMP expression levels ranging from 0.5 to 5 g AMP/kg FW (Figure 5a,b), and for an expression level of 1 g AMP/kg FW over a range of AMP production levels from 100 kg AMP/year to 1,000 kg AMP/year (Figure 5c,d). Note that in all cases, the unit operations were resized to meet the design requirements. COGS decreases with diminishing returns as a function of expression level, as can be seen in Figure 5. To illustrate this point, consider that an increase of expression level from 0.5 to 1 g/kg FW results in $4.43/g decrease in COGS, while an increase from 4 to 5 g/kg FW results in $0.22/g decrease in COGS. These changes are equivalent to 39% and 6% reductions, respectively. Also note that at low expression levels the upstream operating costs contribute more to the COGS, whereas at high expression levels downstream operating costs contribute more to the COGS. This is reasonable because the number of plants per batch will increase as expression level decreases, thus requiring more soilless growth media, seeds, and nutrients. CAPEX follows a similar trend with expression level; however, the downstream process is the main contributor to CAPEX, except for very low expression levels (less than 0.5 g/kg FW). The majority of COGS and CAPEX variation with expression level is attributable to upstream processing, with downstream process costs remaining fairly consistent over the range of expression levels considered.

COGS also decreases with diminishing returns as a function of yearly production capacity. Downstream processing is the main contributor to COGS at low production levels while upstream processing is the main contributor at high production levels; at 100 kg/year, downstream processing represents 64% ($8.51/g) of the COGS, while at 1,000 kg/year the contribution is reduced to 35% ($2.15/g) of the COGS. Within the given parameter range for expression level and production capacity, COGS shows a higher sensitivity to expression level.

**Figure 6:**
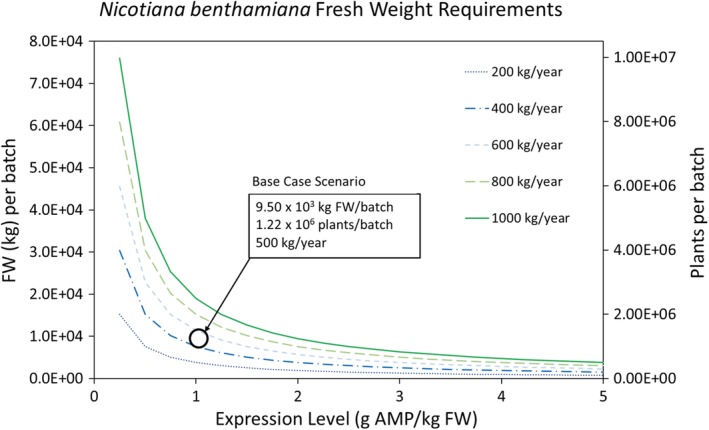
*Nicotiana benthamiana* plant fresh weight (FW) as a function of expression level and yearly production of antimicrobial product (AMP). The base case scenario of 1 g AMP/kg FW expression and 500 kg yearly production requires 9.50 × 10^3^ kg FW, which translates into 1.22 × 10^6^ plants, per batch

Figure 6 shows *N. benthamiana* FW per batch as a function of expression level and yearly production demand. As expected, biomass requirements are reduced at higher expression levels and lower yearly production demand. Variation in the expression level has a higher impact on biomass requirements for higher yearly production demands. At all yearly production levels, significant diminishing returns for increases to expression level are evident within the selected range expression level.

### 3.5 Alternative scenario analysis

The nicotine‐free *S. oleracea* scenario produces 500 kg AMP/year at 1 g AMP/kg FW with 66% product recovery and 63% purity formulation (Table S4). Manufacturing batches require ~10% fewer plants than the base case at 1.08 million *SS. oleracea* plants/batch, and a correspondingly lower plant inventory of 11.1 million plants. The upstream processing duration remains consistent with the base case, while the downstream processing time is reduced to 0.67 days after removal of the nicotine clearance chromatography step of the base case scenario. The *S. oleracea* manufacturing facility requires $46.5 million CAPEX and $2.50 million/year OPEX. In this scenario, AMPs are manufactured at a COGS of $4.92/g.

The field‐grown *N. tabacum* scenario produces 500 kg AMP/year at 1 g AMP/kg FW with 58% product recovery and 92% purity formulation (Table S6). There are 63 manufacturing batches yearly of 13,900 *N. tabacum* plants per batch within the late March to late October growing season of the US Midwest/South. The lower number of plants is because of the much larger size of field grown *N. tabacum* plants compared with indoor grown *N. benthamiana* plants. The total inventory during steady‐state operation is 619,000 plants. The upstream processing duration is 88.4 days, and the larger batches increase the downstream processing time to 1.08 days per batch. The *N. tabacum* manufacturing facility, including dedicated outdoor field equipment for transgenic handling, requires $27.5 million CAPEX and $1.51 million/year OPEX. We have neglected labor costs associated with overseeing environmental release of transgenic material, the United States Department of Agriculture (USDA) Biotechnology Regulatory Services (BRS) regulatory application, and routine USDA Animal and Plant Health Inspection Service (APHIS) inspections. In this scenario, AMPs are manufactured at a COGS of $3.00/g.

A comparison of the capital investment, production costs, and AMP COGS for the *N. benthamiana* base case, nicotine‐free *S. oleracea*, and field‐grown *N. tabacum* scenarios is shown in Table 2.

**Table 2:** A comparison of capital expenditures (CAPEX), operating expenditures (OPEX), and cost of goods sold (COGS) for the three different studied plant‐based antimicrobial product (AMP) production scenarios

Parameter	Unit	Section	*Nicotiana benthamiana* indoor growth (base case)	*Spinacia oleracea* indoor growth	*Nicotiana tabacum* field grown
CAPEX	$ million	Upstream	19.1	19.1	1.30
		Downstream	31.0	27.4	26.2
		Total	50.1	46.5	27.5
OPEX	$ million/year	Upstream	2.01	1.79	0.280
		Downstream	1.43	0.711	1.23
		Total	3.44	2.50	1.51
COGS	$/g AMP	Upstream	4.02	3.52	0.555
		Downstream	2.86	1.40	2.45
		Total	6.88	4.92	3.00

## 4 DISCUSSION

### 4.1 Facility operation of base case scenario

A greenfield single‐product biomanufacturing facility was chosen to reflect the current whole plant protein biomanufacturing environment in the United States. There is significant, yet limited, existing manufacturing capacity, most of which is positioned for pharmaceutical‐grade production. For smaller annual production demands (<300 kg product/year), a single‐ or multi‐product contract manufacturing organization (CMO) model would also be viable. These trends are also reflected globally.

The yearly production was determined to meet the demand of a projected market share anticipated for a product of this nature (unpublished data). The number of yearly batches was determined to fully utilize upstream plant growth capacity while leaving idle time for downstream equipment, which is likely to require more maintenance. Future work could include optimization of the plant inventory size, and thus batch size, to maximize the discounted cash flow rate of return over the project lifetime. The optimization will need to identify a balance in the fluctuation of equipment‐associated CAPEX and labor‐ and utility‐associated OPEX for both the upstream and downstream.

The low‐purity requirement of the AMP at 92% is associated with the selection of plant‐based production and is a distinct advantage over traditional production platforms for food safety applications. Leafy plant extracts are safe for consumption and routinely consumed as a staple of human diet; when the impurities of the host organism are Generally Recognized as Safe (GRAS) for consumption, there is considerably lower burden on downstream processing. The major focus is redirected from product application safety to product stability and functionality in the presence of the host impurities. Depending on the application rate and consumer consumption, we expect that formulations of 50–95% purity could be employed. Therefore, this analysis represents an upper bound for the anticipated production costs.

*Nicotiana benthamiana* has been developed as an efficient recombinant protein expression platform. Except for nicotine and traces of anabasine, the *N. benthamiana* leaf constituents are considered safe for human consumption. Therefore, the processing and quality control are centered on host alkaloid reduction. Processing with a single‐cation exchange column can provide log reduction in the nicotine level in the product stream to <10 ng nicotine/mg TSP in the formulated product. Based on this reduction, the maximum daily intake of nicotine from the use of colicin as a food safety AMP would be much lower than is encountered in everyday consumption of *Solanaceae* plants such as peppers, tomatoes, or potatoes.^15^

#### 4.1.1 Upstream processing

Vertical farming is just beginning to receive commercial interest as an agricultural solution for year‐round, locally grown produce free of pesticides. As the vertical farming industry continues to gain traction, technological advances and process intensification will arise that substantially reduce manufacturing costs for both vertical farming of agricultural crops and plant molecular farming. For example, efficient capture and recirculation of water lost to transpiration (up to 99% water absorbed by roots) will greatly reduce water requirements. Continued development of light emitting diode (LED) systems is expected to further improve growth rates, which should help reduce CAPEX, utility costs, and plant growth cycle time.

Based on this current techno‐economic analysis, advances in plant substrate processing strategies have particularly high potential for economic gain. Soilless plant substrate represents 41% of the overall OPEX in the *N. benthamiana* base case scenario. A single reuse of the soilless plant substrate prior to disposal would lower the overall OPEX by ~21% in the reduction of the cost of consumables. Reuse of the plant substrate can be achieved by either regrowth of harvested plants or a second round of seeding to generate new plants. In the former situation, manufacturing cost reductions would also include those associated with seeding and tray cleaning operations.

#### 4.1.2 Downstream processing

Future model optimization could be explored to investigate the impact of lot pooling on COGS. However, lab data should be performed in tandem to support the choice of lot pooling in the manufacturing scheme and the storage conditions. It is well known that proteases present in the leafy plant extract can degrade protein molecules of interest.^41^ Proteases present in the leafy plant extract should be removed or inactivated to reduce proteolytic cleavage and maintain product recovery in the case of a hold step prior to lot pooling.

### 4.2 Economic analysis of base case

Techno‐economic analysis provides critical information at all stages of a project's lifetime. Efficiency of internal research and development in biotechnology companies has suffered in recent years.^42^ Techno‐economic analysis is a useful tool for improving this efficiency through identification of key economic‐influencing parameters and insights into the commercialization potential of the proposed technology. This preliminary analysis provides early indicators of success potential and reduces the risk of investment for key stakeholders. Furthermore, scenario analysis can guide research and development prioritization to maximize return on investment. In the base case model of this study, a change in the expression level from 0.5 to 1 g AMP/kg FW resulted in 20‐fold greater COGS savings than from 4 to 5 g AMP/kg FW. This knowledge makes it clear that there is a significant economic incentive to improve expression levels, but only up to a point. Refinement of the analysis with pilot‐scale data further strengthens the analysis and provides perspective to inform future scale‐up work. At the stage of commercial production, techno‐economic analyses can provide essential insights into areas such as scheduling, vendor contracts, continuous improvement, and process intensification.

### 4.3 Purchase price sensitivity analysis

Analysis of these individual factor sensitivities provide a preliminary framework for understanding expected bounds of manufacturing costs. It can also serve as a prioritization tool for vendor selection when considering larger, multimaterial contracts, as well as with research and development efforts.

This analysis could be strengthened to include a forecasting capacity in future work by integrating market analyses to weight each level of factor variation with a likelihood based on predictive market data. From this information, one could establish an anticipated range of COGS based on key cost factors to holistically define uncertainty and risk.

### 4.4 Yearly production demand and expression‐level analysis

Within the given parameter range for expression level and yearly production volume, COGS is more strongly impacted by the expression level. This behavior is specific to the defined parameter ranges, which were selected based on anticipated needs and expectations. In this study, we assumed that raw material and consumable resource purchase costs per unit are independent of yearly amount purchased. As yearly production increases, economies of scale dictate that the material unit price will decrease. This becomes a more important consideration when evaluating COGS over a wide yearly production range.

Figure 5 shows similar behaviors for changes in total COGS with expression level and yearly production. However, there is a dissimilar behavior in the upstream versus downstream contributions to COGS over the parameter range. Varying expression level largely influences the upstream processing COGS, while varying yearly production largely influences the downstream processing COGS. The low downstream COGS sensitivity to expression level is mainly attributed to two items. The main reason is that the costly downstream operations (e.g., chromatography) are economically dependent on AMP quantity rather than on stream composition. Additionally, we chose to conservatively fix AMP recovery in the downstream, regardless of expression level. The low upstream COGS sensitivity to yearly production is because of the approximately linear scalability of the production platform. This is a main advantage of plant‐based production that makes the scale‐up from lab to commercial scale considerably simpler and faster than traditional bioreactor‐based production platforms.^43^ As yearly production changes, the upstream processing scales in an approximately linear fashion for a given processing strategy. However, one could anticipate that scaling to even higher yearly production could enable higher efficiency upstream processing strategies and thus improve the scaling dynamics of upstream economic contributions.

### 4.5 Alternative scenario analysis

The nicotine‐free *S. oleracea* scenario provides insight into the manufacturing costs associated with nicotine clearance. There are minor differences in plant growth and harvest operations, but the majority of upstream COGS reduction is because of higher product recovery and thus lower biomass requirements for a given yearly production level. Higher product recovery is attributed to the removal of the nicotine clearance chromatography step present in the *N. benthamiana* base case scenario, as illustrated in Figure 2. The smaller batch size and simpler downstream processing as compared to the *N. benthamiana* base case scenario result in a 26% reduction in the downstream cycle time and 37% reduction in downstream labor costs, yielding a COGS of $4.92/g AMP.

The field‐grown *N. tabacum* scenario results in the lowest COGS of $3.00/g AMP, providing reasonable justification to pursue this manufacturing process. However, our assumptions do not account for potential upstream difficulties associated with product expression consistency, greenhouse growth, and transplantation of seedlings (direct field seeding is assumed) or crop loss because of adverse weather events throughout the growing season, nor do they account for the downstream difficulties associated with removal of the more viscous *N. tabacum* host leaf impurities. Future work to experimentally support key assumptions of field growth could add higher confidence and value to this alternative scenario. Additionally, the current growth strategy is based on tobacco production as a commodity good; there may be a different growth strategy that is optimal for recombinant protein production (e.g., increased planting density with reduced time to harvest and higher number of batches per year). It is worth noting that this manufacturing process is expected to scale especially well. In our model, we assume that dedicated personnel and upstream equipment are required for transgenic handling. At an annual production level of 500 kg AMP, this results in 17% upstream equipment utilization. This means that as the yearly production demand increases, we expect marginal increases to upstream CAPEX and OPEX. As such, we expect upstream‐related COGS to reduce dramatically with increases in yearly production demand.

### 4.6 Cost of use

Biotic food sanitizers can be used in a variety of applications to augment traditional food sanitizing treatments against specific high‐risk pathogens. Given the differences in food safety practices among food products, it can be difficult to measure the cost of use as a single value. Instead, we focused our discussion on cost of use calculations with application rates representative of AMP use—colicins for control of *E. coli* on red meats. We chose to investigate this example at several points along beef processing: animal washing, post‐slaughter carcass cleaning, and meat product protection. We anticipate an application rate of 2–10 ppm AMP in water for animal and carcass wash or 2–10 mg AMP per kg meat product. It should be pointed out that, according to the recently published paper of Hahn‐Löbmann et al. in 2019, the application rates of salmocins, *Salmonella* ‐derived bacteriocins, could be up to 10 times lower because of the higher potency of salmocins.^44^

**Figure 7:**
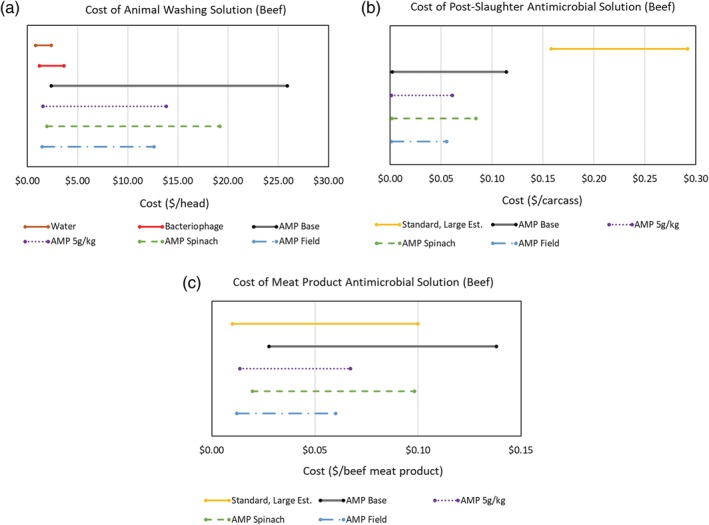
Cost of use estimates for antimicrobial protein (AMP) on beef based on expected application rates for (a) animal washing, (b) post‐slaughter carcass spray, and (c) meat products. Values are compared to relevant product pricing and across multiple manufacturing production strategies: The base case *Nicotiana benthamiana*, the highest expected expression *N. benthamiana* (5 g AMP/kg fresh weight), the nicotine‐free *Spinacia oleracea*, and the field‐grown *Nicotiana tabacum* scenarios

Figure 7 shows the cost of use estimates for select techno‐economic scenarios modeled in this study compared to relevant standard sanitizing treatments. Cost of use assumptions and a sample calculation of those performed to generate the cost of use estimates can be viewed in Table S7 and Calculation S1, respectively. In all three points of intervention, AMP application cost ranges are below or overlapping those of standard treatments. Additional information is needed on application rates and spray volume used in animal washing to reduce AMP cost of use range and increase confidence in cost comparison to standard treatments. On the other hand, AMP cost of use ranges for treatment of meat product overlap significantly with standard interventions, indicating comparable costs. Finally, the AMP cost of use ranges for post‐slaughter carcass cleaning suggest that the use of AMP at this beef processing juncture has the potential to be substantially lower in cost than standard treatments.

## 5 CONCLUSIONS

Current food safety practices, although largely effective, result in foodborne illnesses that impose a $14 billion annual burden on the US healthcare system. As the looming prevalence of antibiotic resistance grows, so will the impact of foodborne illnesses. The need for protection against foodborne pathogens is only increasing.

Reports as far back as 20 years ago acknowledge that areas of the food industry like the meat sector will need to absorb additional costs to improve food safety levels.^45^ We investigated bacteriophage‐derived lysins and bacteria‐derived AMPs to explore the capacity of this class of biotic sanitizers to improve food safety levels in the cost‐sensitive food industry. Although previous studies illustrate the efficacy of AMPs, in this study, we performed a techno‐economic analysis of plant‐based production of AMPs to better understand the commercialization potential of products produced using this platform. Our analysis predicts a $6.88/g AMP COGS for the base case scenario, $4.92/g for the nicotine‐free *S. oleracea* scenario, and $3.00/g for the field‐grown *N. tabacum* scenario. We also evaluated the sensitivity of the base case COGS to changes in purchase price, expression level, and yearly production. In doing so, we identified economic “hot spots,” which include the large contribution of the soilless plant substrate (41.2% of annual operating costs; Figure 4b) and downstream labor‐dependent costs (18.5%). The cost of use analysis indicates that AMPs are projected to de‐risk foodborne disease in beef processing as supplemental sanitizing treatments at only minor economic perturbation across several key processing junctures. It is expected that other food processing operations would yield similar benefits.

This techno‐economic analysis of plant‐based production of AMPs is focused on manufacturing costs and the implications for application costs. In developing this model and analysis, we have identified several areas of importance for future analysis, for example, consideration of avoided costs associated with the prevention of food disease and illness. An example of a major avoided cost is that associated with food recall, which includes impact to brand image and loss of sales. A cost–benefit model that includes these avoided costs may provide more complete insights into AMPs as a food sanitizing treatment. In addition, there are social, cultural, and behavioral factors that can impact food safety that are not considered in this economic analysis.

In our analysis, we describe plant‐based production of AMPs as a food processing aid. A direct evaluation of traditional manufacturing platforms, such as mammalian cell suspension culture and bacterial fermentation, as alternative scenarios would be a valuable future contribution. To our knowledge, there are no existing direct comparisons of whole plant, microbial fermentation, and mammalian cell culture platforms in the literature. Future work to compare AMP manufacturing in different locations would also add insight into the geographical and national sensitivity of AMP manufacturing process costs.

We compare three host plant batch production models in our analysis, all with different manufacturing processes. A valuable future analysis would be to additionally compare alternative operational modes for a single host plant. Continuous manufacturing is a nascent biotechnology process intensification trend that describes processing of a target molecule from raw materials to final product without any hold steps in a continuous flow process. This contrasts with the more traditional batch manufacturing investigated in this analysis, in which discrete batches are processed at time intervals. It is generally accepted that continuous manufacturing reduces facility footprint, buffer usage, and equipment sizing as compared to batch manufacturing. To date, there are no publications of continuous manufacturing using plant‐based production. We anticipate that plant‐based production is a favorable platform for continuous manufacturing, which can reduce CAPEX costs through the replacement of large steel vessels with small disposable containers; whole plant production does not require disposable containers, as the plant itself functions as the bioreactor. A techno‐economic analysis comparing these two manufacturing modes will provide additional insight into the economics of plant‐based production.

## DISCLOSURE OF INTEREST

The outcomes of this study are personal views of independent authors (M.M., Y.G., D.T., S.H., A.G., S.N., and K.M.). The outcomes do not reflect any financial or commercial interest of the University of California, Davis, Nomad Bioscience GmbH, or DT Consulting Group.

## Supporting information


**Appendix S1**: Supplementary information
