# Supplementary material for: Techno‐economic analysis of a plant‐based platform for manufacturing antimicrobial proteins for food safety
Source: Biotechnol Prog. 2019 Sep 11;36(1):e2896. doi: 10.1002/btpr.2896 (PMC7027456; doi:10.1002/btpr.2896)
Supplement: Supplementary file 1 — Appendix S1: Supplementary information [file BTPR-36-e2896-s001.docx]

SUPPLEMENATRY INFORMATION


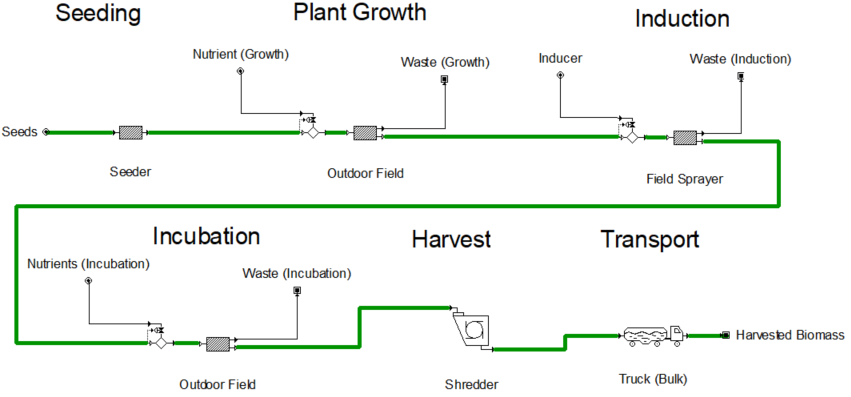


**Figure S1:** Visual representation of the *Nicotiana tabacum* alternative scenario upstream processing SuperPro Designer® model.

**
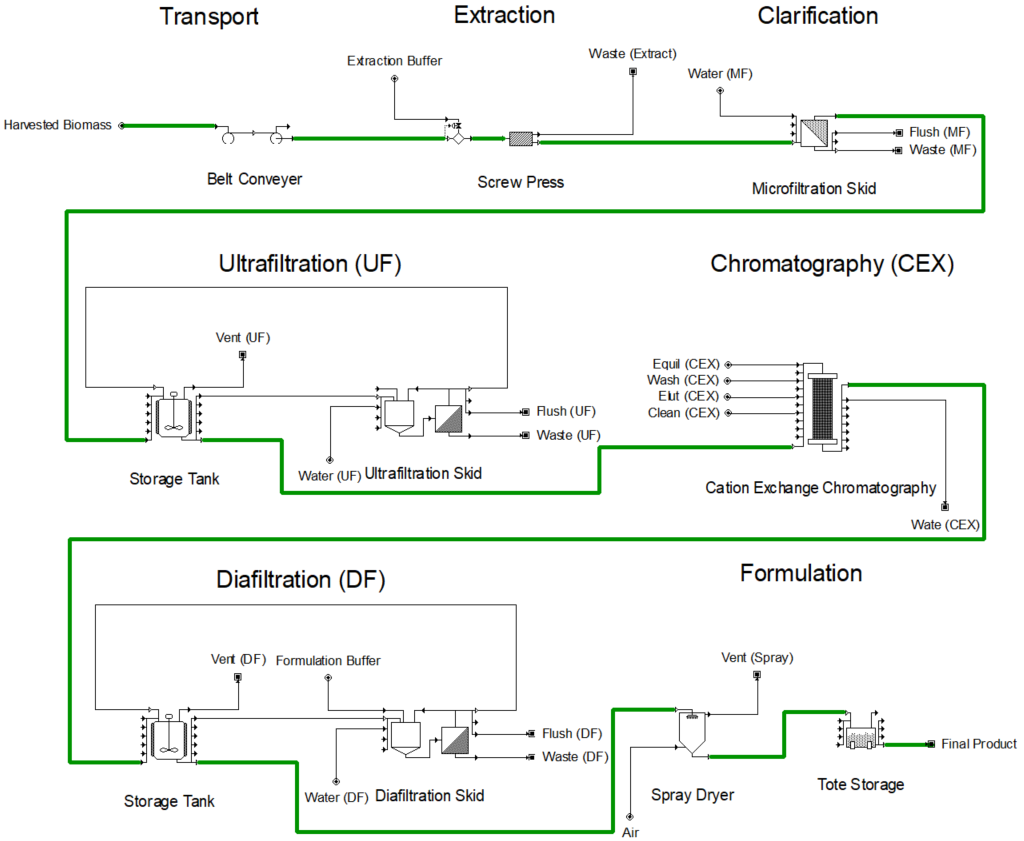

Figure S2:** Visual representation of the *Nicotiana tabacum* alternative scenario downstream processing SuperPro Designer® model.

| Input Parameter | Value |
| --- | --- |
| Yearly Production Demand | Per year: 500 kg antimicrobial product  Per batch: 9.50 kg antimicrobial product |
| Germination Efficiency | 95% |
| Expression Level | 1 g antimicrobial product/kg biomass FW; 10% total soluble protein |
| Downstream Recovery | 97% Screw Press  85% Microfiltration  85% Ultrafiltration  87% Chromatography  94% Diafiltration  100% Spray Dry  58% TOTAL |
| Product Purity | 92% |

**Table S1:** Facility design assumptions for the base case scenario model. Values are based on working process, in general. Expression level is based on Werner et al. 2011.

| Input Parameter | Purchase Price Per Year | Quantity Per Year |
| --- | --- | --- |
| Upstream Operator | $159,000  Total Labor Cost = $46/hr  95% Time Utilization | 3,450 labor hours |
| Downstream Operator | $507,000  Total Labor Cost = $81/hr  60% Time Utilization | 6,300 labor hours |
| Local Power | $244,000  Rate = $0.0548/kW-h | 4,450,000 kW-h |
| Local Water | $1,000  Rate = $0.809/MT | 1,180,000 L |
| Steam | $1,070  Rate = $12/MT | 89.0 MT |
| Chilled Water | $381  Rate = $0.4/MT | 952 MT |
| Plant Seed | $117,000  Rate = $9.5/g | 12,400 g  (1 gram = 9500 seed) |
| Soilless Plant Substrate | $1,420,000  Rate = $12.8/1000 plants | 111,000 items |
| Nutrient Solution | $65,000  Rate = $0.05/L | 1,300,000 L |
| Ethanol, 3% (w/w) | $172  Rate = $0.02/L | 7,400 L |
| Extraction Buffer | $64,000  Rate = $0.16/L | 407,000 L |
| Equilibration Buffer | $32,000  Rate = $0.15/L | 204,000 L |
| Wash Buffer | $5,000  Rate = $0.04/L | 127,000 L |
| Elution Buffer | $47,000  Rate = $0.35/L | 128,000 L |
| Cleaning Buffer | $11,000  Rate = $0.14/L | 77,000 L |
| Formulation Buffer | $4,000  Rate = $0.07/L | 63,000 L |
| HNO_3_, 0.5% (w/w) | $1,000  Rate = $2.61/MT | 400 MT |
| NaOH (0.5 M) | $54,000  Rate = $0.14/L | 382,000 L |
| Plastic Bag (1 L) | $240  Rate = $0.20/unit | 1,200 units |

**Table S2:** Model operating expenditure input purchase prices and quantity used for the base case scenario model. Values are based on working process, in general. Local power and water values were obtained from Owensboro, Kentucky municipal services.

| Input Parameter | Value |
| --- | --- |
| Facility-Dependent Costs | Basis: maintenance cost  Neglected: depreciation, insurance, local taxes, factory expense |
| Maintenance Cost | Basis: % equipment purchase cost (section dependent) |
| Laboratory / Quality Assurance / Quality Control | Basis: % total labor cost (section dependent) |
| Labor Cost | Basis: total labor cost (TLC) = basic labor rate x (1 + benefits(0.4) + supervision(0.2) + supplies(0.1) + administration(0.6)) |
| Labor Types | Upstream Operator   - Basic rate = $20.00/hr; TLC = $46.00/hr - Time Utilization = 95%   Downstream Operator   - Basic rate = $35.00/hr; TLC = $81.00/hr - Time utilization = 60% |
| Direct Fixed Capital (DFC) | Basis: 1.2 x listed purchase equipment cost (20% for unlisted equipment) + section-dependent factor (see below)  Upstream  + 3.0 x listed purchase equipment cost (direct and indirect costs, e.g. piping, instrumentation)  Downstream  + 6.0 x listed purchase equipment cost (direct and indirect costs, e.g. piping, instrumentation) |
| Working Capital (WC) | Basis: 30 days raw materials, labor, utilities, waste treatment |
| Startup Costs | Basis: % DFC (section dependent)  Neglected: upfront research and development, upfront royalties, land purchase cost |
| Income Tax | Basis: 40% |
| Project Financing | Basis: 0% debt; no loans for DFC or WC |

**Table S3:** Operating expenditure and capital expenditure bases for the base case scenario model. Values are based on working process, in general.

| Parameter | | Value | | Units |
| --- | --- | --- | --- | --- |
|  |  | *Nicotiana benthamiana* Indoor Growth  (Base Case) | *Spinacia oleracea* Indoor Growth |  |
| Facility Design | AMP Recovery | 58 | 66 | % |
|  | AMP Purity | 92 | 63 | % |
| Plant Cultivation | Growth facility cost | 500 | 500 | $/m^2^ |
|  | Growth space utilization | 70 | 70 | % |
|  | Growth space layers | 3 | 3 | layers |
|  | Seed mass | 1.05E-04 | 1.05E-04 | g/seed |
|  | Seed mass per batch | 1.36E+02 | 1.20E+02 | g seed/batch |
|  | Plants per batch | 1.22E+06 | 1.08E+06 | plants/batch |
|  | Plant density | 6.24E+02 | 6.00E+02 | plant/m^2^ |
|  | Tray area | 1.50E-01 | 1.73E-01 | m^2^/tray |
|  | Plants per tray | 9.40E+01 | 1.04E+02 | plant/tray |
|  | Trays per batch | 1.30E+04 | 1.04E+04 | trays/batch |
|  | Plant growth rate | 1.89E-04 | 1.86E-04 | kg/day/plant |
|  | Leaf biomass, harvest | 4.29E-03 | 3.85E-03 | leaf kg/plant |
|  | Aerial biomass, harvest | 7.77E-03 | 7.74E-03 | aerial kg/plant |
|  | AMP per plant, harvest | 7.77E-06 | 7.74E-06 | kg AMP/plant |
|  | Plant inventory | 1.25E+07 | 1.11E+07 | plants/facility |

**Table S4:** *Nicotiana benthamiana* base case and *Spinacia oleracea* alternative scenario assumptions for cultivation inputs. Facility design parameters that are different between the two scenarios are also highlighted. All input values are based off working process knowledge.

| Buffer | Constituents | Quantity |
| --- | --- | --- |
| Extraction buffer (pH 4.0) | 200 mM glycine  200 mM sodium acetate  200 mM sodium phosphate  200 mM sodium bicarbonate  200 mM sodium chloride | Location: screw press  4,510 L/batch |
| Equilibration buffer (pH 4.0) | Same composition as extraction buffer | Location: chromatography  2,260 L/batch |
| Wash buffer (pH 4.0) | 50 mM sodium chloride  2.5 mM sodium di-hydro phosphate | Location: chromatography  1,410 L/batch |
| Elution buffer (pH 7.8) | 1 M sodium chloride  50 mM sodium di-hydro phosphate | Location: chromatography  1,410 L/batch |
| Cleaning buffer (pH 13.5) | 0.5 M NaOH | Location: chromatography  848 L/batch |
| Formulation buffer (pH 7.4) | Phosphate-buffered saline (PBS)  137 mM sodium chloride  2.7 mM potassium chloride  10 mM sodium hydro phosphate  1.8 mM potassium di-hydro phosphate | Location: diafiltration  690 L/batch |

**Table S5:** Buffer composition and usage for the base case scenario downstream processing model. Values are based on working process, in general. Extraction buffer composition is based on Azzoni et al. 2002.

| Parameter | | Value | Units |
| --- | --- | --- | --- |
| Facility Design | Yearly batches | 63 | batches/year |
|  | Growing season | 214 | days/year |
|  | AMP purity | 92 | % |
|  |  | Basis: 1.2 x listed purchase equipment cost (20% for unlisted equipment) + section-dependent factor (see below)  Upstream  + 1.0 x listed purchase equipment cost (direct and indirect costs, e.g. piping, instrumentation)  Downstream  + 6.0 x listed purchase equipment cost (direct and indirect costs, e.g. piping, instrumentation) |  |
| Plant Cultivation | Irrigation equipment | 0.247 | $/m^2^ |
|  | Irrigation operation | 0.099 | $/m^2^/year |
|  | Tobacco production | 0.334 | $/m^2^/year |
|  | Plant density | 1.30E+01 | plant/m^2^ |
|  | Aerial biomass, harvest | 1.00E+00 | aerial kg/plant |
|  | Plant growth rate | 1.12E-02 | kg/day/plant |
|  | Plant growth time | 82 | days |
|  | AMP per plant, harvest | 1.00E-03 | kg AMP/plant |
|  | Plants per batch | 1.39E+04 | plants/batch |
|  | Seed mass per batch | 1.54E+00 | g seed/batch |
|  | Plant inventory | 6.19E+05 | plants/field |
|  | Land turnaround time | 3 | days |
|  | Field utilization | 95 | % |

**Table S6:** Field-grown *Nicotiana tabacum* alternative scenario unique inputs from the base case scenario. All input values are based off Tusé et al. 2014 and working process knowledge. AMP, antimicrobial product.

| Parameter | | Value | | | Units |
| --- | --- | --- | --- | --- | --- |
|  |  | Low | Midpoint | High |  |
| Large Establishment Antimicrobial Costs (Post-Slaughter) | Organic Acids | 0.158 | 0.175 | 0.193 | $/head |
|  | Peracetic Acid | 0.239 | 0.265 | 0.292 | $/head |
| Water Use (Post-Slaughter) | | 0.05 | 0.40 | 0.75 | L/head |
| Bacteriophage Cost  (Animal Wash Only) | | 1.22 | 2.42 | 3.64 | $/head |
| Water Use (Animal Wash) | | 57 | 114 | 170 | L/head |
| Large Establishment Antimicrobial Costs  (Meat Product) | Undisclosed treatment | 0.01 | 0.05 | 0.10 | $/kg meat |

**Table S7:** Assumptions for AMP cost of use estimations in beef processing, based on information provided by the USDA (Noyes et al. 2015). Meat product antimicrobial costs are based on working process knowledge and unpublished market analyses.

**Calculation S1:** Cost of use estimates for select techno-economic scenarios

Costs of relevant sanitizing applications for animal washing and post-slaughter carcass cleaning are based on 2015 values prepared by the United States Department of Agriculture (USDA) (Noyes et al. 2015), while reference application pricings for red meat products are based on unpublished market analysis. Carcass size is based on previously reported values (Ripoll et al. 2016). Liquid volume required to wet a carcass volume with AMP solution (15 – 45 gallons/head) is based on working process knowledge and values in literature (A. Estes Reynolds 2005). The AMP COGS is expected to represent 50% of the selling price.

Example Cost of Animal Washing Solution calculation for the AMP base case using the lower bound AMP concentration (2 ppm) and lower bound water use (15 gallons/head) for the animal wash point of intervention:

$$Production cost of AMP\left( \frac{\$6.88}{g AMP} \right)\times selling price conversion \left( 2 \right)\times AMP working concentration \left( \frac{0.002 g AMP}{L water} \right) \times application rate \left( \frac{56.8 L water}{head} \right)+ purchase price of water \left( \frac{\$0.014}{L water} \right)\times application rate \left( \frac{56.8 L water}{head} \right)= AMP sanitizing treatment cost (\frac{\$2.36}{head})$$
